# From Infancy to Childhood: A Comprehensive Review of Event- and Task-Related Brain Oscillations

**DOI:** 10.3390/brainsci14080837

**Published:** 2024-08-20

**Authors:** Esra Ünsal, Rümeysa Duygun, İrem Yemeniciler, Elifnur Bingöl, Ömer Ceran, Bahar Güntekin

**Affiliations:** 1 Department of Neuroscience, Graduate School of Health Sciences, Istanbul Medipol University, 34810 Istanbul, Turkey; esra.unsal@std.medipol.edu.tr (E.Ü.); rumeysa.duygun@std.medipol.edu.tr (R.D.); irem.yemeniciler@std.medipol.edu.tr (İ.Y.); elifnur.bingol@std.medipol.edu.tr (E.B.); 2 Neuroscience Research Center, Research Institute for Health Sciences and Technologies (SABITA), Istanbul Medipol University, 34810 Istanbul, Turkey; 3 Department of Biophysics, School of Medicine, Istanbul Medipol University, 34810 Istanbul, Turkey; 4 Department of Pediatrics, School of Medicine, Istanbul Medipol University, 34810 Istanbul, Turkey; oceran@medipol.edu.tr

**Keywords:** infant, childhood, electroencephalography, event-related oscillations, task-related oscillations

## Abstract

Brain development from infancy through childhood involves complex structural and functional changes influenced by both internal and external factors. This review provides a comprehensive analysis of event and task-related brain oscillations, focusing on developmental changes across different frequency bands, including delta, theta, alpha, beta, and gamma. Electroencephalography (EEG) studies highlight that these oscillations serve as functional building blocks for sensory and cognitive processes, with significant variations observed across different developmental stages. Delta oscillations, primarily associated with deep sleep and early cognitive demands, gradually diminish as children age. Theta rhythms, crucial for attention and memory, display a distinct pattern in early childhood, evolving with cognitive maturation. Alpha oscillations, reflecting thalamocortical interactions and cognitive performance, increase in complexity with age. Beta rhythms, linked to active thinking and problem-solving, show developmental differences in motor and cognitive tasks. Gamma oscillations, associated with higher cognitive functions, exhibit notable changes in response to sensory stimuli and cognitive tasks. This review underscores the importance of understanding oscillatory dynamics to elucidate brain development and its implications for sensory and cognitive processing in childhood. The findings provide a foundation for future research on developmental neuroscience and potential clinical applications.

## 1. Introduction

Neuron migration and differentiation, followed by the formation of abundant synaptic connections and specialized neural networks, are some of the fundamental events that occur during the developmental process [1,2,3]. This process is the most intensive within layer IV of the cortex, which is known as a major recipient layer of thalamocortical projection. However, the process of address selection [4] by thalamocortical input promotes elaboration of the cortical circuitry. The elaboration of thalamocortical circuitry and interaction with sensory input strengthens sensory-driven activity, further shaping fine connectivity within the cortical columns [5]. MRI studies have provided compelling insights into the structural changes occurring in the brain during childhood and adolescence. The maturation of the cortex parallels cognitive milestones in human development. Regions supporting primary functions, such as motor and sensory systems, mature earliest, while temporal–parietal areas associated with basic language skills and spatial attention develop subsequently. Higher-order integration regions, such as the prefrontal and lateral temporal cortices, are among the last to mature. The physiological basis for this maturation is supported by mechanisms of experience-dependent synapse formation and dendritic arborization [6,7,8,9]. As growth and development progress, the electroencephalography (EEG) exhibits a trend towards continuity, although slow activity transients are present, indicating functional immaturity [10,11].

The EEG is used to record the electrophysiological activity generated by neural networks and is thus extremely sensitive to changes in their organization throughout developmental processes, as well as in cases of pathological disturbances. Moreover, these generated brain oscillations are considered the “functional building blocks” of sensory and cognitive processes [12]. Hence, EEG brain oscillations of different age and disease groups demonstrate varying characteristics based on the maturity and integrity of their sensory and cognitive processing systems [13,14]. Erol Başar conceptualized how the brain generates responses to external and internal stimuli and its dynamic response mechanisms through the framework of susceptibility rules. Within this framework, it is articulated that the brain responds at different frequencies depending on the types of stimuli and cognitive processes. Oscillatory activity can be modulated by the type of stimulus, whether it is visual, auditory, or tactile. Similarly, the oscillatory activity obtained varies depending on the type of cognitive function, such as attention, memory, or problem-solving [15].

The most common hypothesis proposed to explain the decrease in the absolute spectral power during development refers to the reduction in gray matter associated with maturation [16,17,18]. In fact, a parallel developmental trajectory between the reduction in cortical thickness and the decrease in spectral power with age has been observed [16]. It has been suggested that the increase from low to high frequencies in the brain oscillations and the increase in the alpha frequency band from childhood to adulthood would be due to the increase in the speed of neural communication, basically produced by the increase in the speed of action potentials due to myelination and/or an increase in the axon diameter [19,20,21,22]. Considering that brain plasticity, which forms the basis of the ability to adapt to environmental conditions, is at its peak in early childhood, it becomes more essential to follow neuronal development processes in childhood [23,24]. EEG brain oscillations are produced randomly by various groups of neural generators oscillating at different frequencies. However, by delivering sensory stimulation, these generators are combined and act together harmoniously. Synchronized activities lead to evoked and induced rhythms. These combined waves undergo filtration to generate event-related brain oscillations spanning a frequency spectrum from 0.5 Hz to 100 Hz, encompassing delta through gamma frequencies. This entails the segmentation of EEG frequencies into distinct bands, including delta (0.5–3.5 Hz), theta (3.5–7 Hz), alpha (8–13 Hz), beta (18–30 Hz), and gamma (30–70 Hz) oscillations [25]. Alpha frequency is in the range of 8–13 Hz, which reflects the essential physiological characteristics of the brain. It occurs in sensory, motor, cognitive, and memory tasks. Furthermore, the differences in the evolutionary and developmental emergence of the alpha frequency are noteworthy in the studies [26,27,28,29]. In a longitudinal investigation focusing on infants’ event-related responses to auditory stimuli, analysis of EEG data showed that infants exhibit a stimulus-evoked neural synchrony pattern similar to adults, characterized by a 1/f distribution. Furthermore, the introduction of the stimulus triggers simultaneous increases in theta, beta, and gamma power [30].

The frequency of brain oscillations generally exhibits a negative correlation with their amplitude, indicating that the amplitude decreases as frequency increases. The amplitude of oscillations is considered to be proportional to the number of synchronously active neural elements, with slower oscillating cell populations composed of more neurons than faster oscillating cells [31,32]. In this context, it is expressed that changes in power related to events (event-related synchronization—ERS and desynchronization—ERD) reflect alterations in the activity of local interactions between principal neurons and interneurons that control the frequency components of the ongoing EEG [31]. The heightened short-term alpha enhancement observed in participants while anticipating a specific sensory stimulus serves as a strong example of power changes related to the event [33]. Oscillations related to events are divided into two categories based on their relationship with stimuli: induced and evoked. Evoked oscillations occur after stimulation and are phase-locked to the stimulus. In contrast, induced oscillations arise without phase-locking to the stimulus [34]. Theta and alpha oscillations play a significant role in attention, memory, and learning processes. Theta oscillations are generally associated with memory and learning, while alpha oscillations are linked to the direction of attention and visual processing. Beta oscillations are related to motor control and task-focused attention, whereas gamma oscillations play a critical role in the integration of cognitive processes. Therefore, the analysis of brain oscillations is an important tool for assessing and understanding individuals’ cognitive abilities [35].

This review aims to identify developmental changes in the characteristics of event-related and task-related oscillations. To this end, oscillatory responses in delta, theta, alpha, beta, and gamma frequency bands will be examined across different developmental periods, starting from infancy to childhood. In this context, examining childhood oscillations will contribute to providing an updated framework for cognitive developmental research by emphasizing the functional relevance of frequency-specific event-related oscillations and networks

## 2. Materials and Methods

The literature search was performed using Medline (PubMed) and Science Direct databases. Keywords included brain oscillation, event-related, and children (or similar words; infants, toddlers, task-related) [36]. The first literature search was conducted in August 2022, with updated searches in August 2023 and October 2023. This article aims to understand the development of event/task-related brain oscillations without any pathological conditions. The articles that comprise the diagnostic groups or cases were excluded.

Eligible studies were identified based on the following criteria:

(a) Inclusion of event- or task-related oscillations in children, toddlers, or infants.
(b) Recording of EEG activity while participants performed a task (i.e., not resting state EEG).
(c) Examination of EEG power, phase, or other time-frequency analyses in response to task events, such as event-related increases or decreases.
(d) No case studies or review articles.
(e) Written in English.

**Figure 1:**
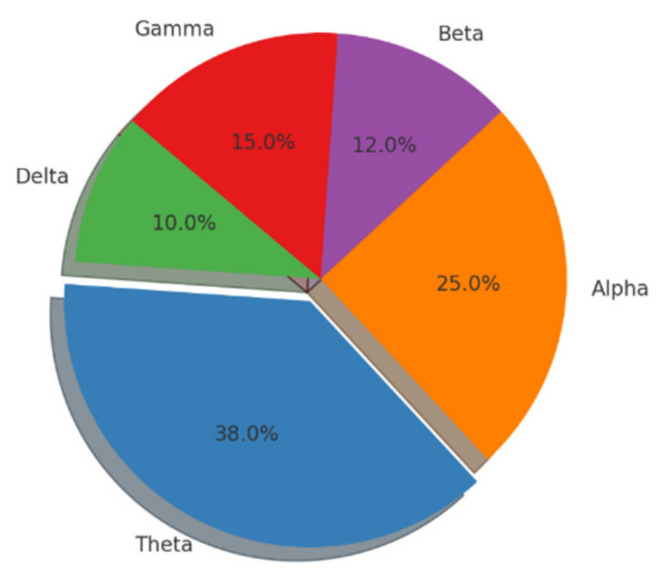
The distribution of event-related EEG studies based on frequencies.

**Figure 2:**
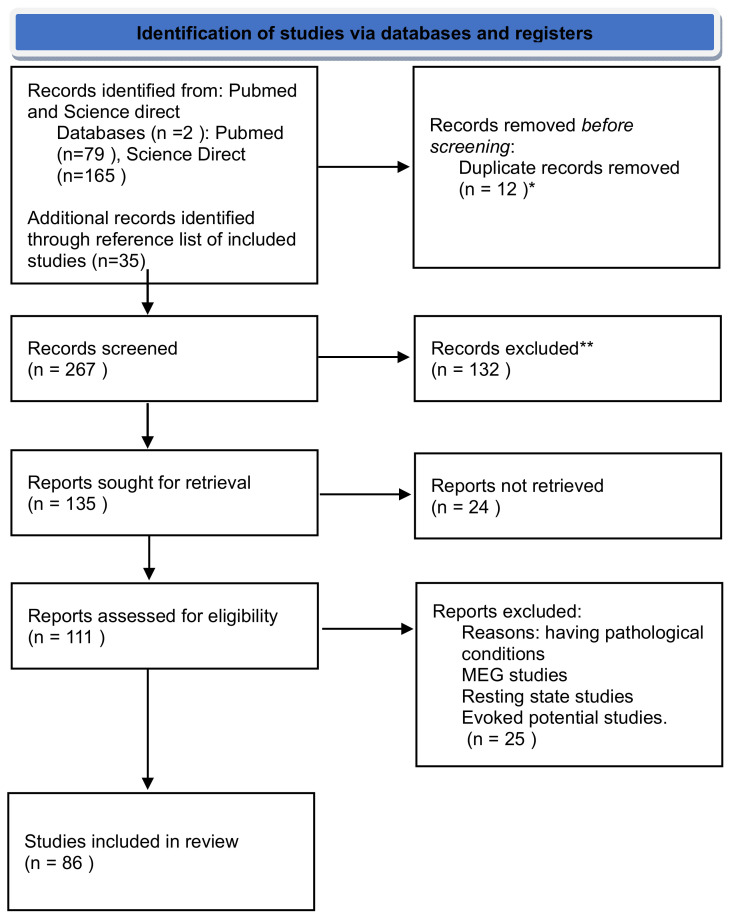
Flow diagram displaying the screening, selection and review process. * The automation tool (Rayyan) was used, indicates how many records were excluded by automation tools. ** Automation tool was used, indicates how many records were excluded by a human.

Articles were imported to the open-source software Rayyan, where duplicate articles were excluded. Ouzzanni and his team developed Rayyan (http://rayyan.qcri.org, accessed on 2 July 2024), a free web and mobile application that accelerates the initial screening of abstracts and titles through a semi-automated process while maintaining a high level of usability [37]. To minimize the risk of bias in the included studies, the first and second authors independently screened titles and abstracts of all studies for eligibility and marked studies as “exclude”, “maybe”, or “include”. All studies marked as “include” or “maybe” by either author were carried forward to the full-text screening, performed independently by the same two authors. Finally, 86 studies meeting all eligibility criteria were selected for inclusion. Theta oscillations are classified as childhood’s most studied frequency bands (Figure 1 and Figure 2).

## 3. Brain Oscillation from Infancy to Childhood

### 3.1. Delta Oscillations: The Foundation of Early Neural Development

As previously acknowledged, delta oscillations in children are scarce. Consequently, there is a need to provide a condensed overview of delta oscillations based on the most extensively researched topics and concepts. The slow wave rhythm, regarding the delta frequency, is predominantly seen during the deepest phases of sleep and significantly diminishes in the wakeful states of adults [38]. The presence of delta activity during wakefulness is often interpreted as an indicator of brain injury or pathological states stemming from neurological harm or psychiatric disorders [39]. In children, delta EEG activity exceeds that of adults due to the ongoing process of cortical maturation, and it is often more pronounced in children who have learning disabilities [40,41]. The study of language and language acquisition in children is a regular focus. The early stages of life are particularly critical, marked by numerous neurological changes that facilitate the acquisition of new abilities, with language being a prominent one. Language acquisition is recognized as involving the processing of both semantic and phonological information, thereby imposing cognitive demands. In our previous review article, which discussed evoked and event-related delta responses, it was observed that heightened delta oscillatory responses in frontal and central brain regions were associated with increased cognitive demands in various tasks [42]. Conversely, heightened delta responses in temporal, parietal, and occipital regions were linked to the perception of facial expressions and the recognition of faces in adults. While a substantial body of research in adults has established connections between event-related delta oscillations and various cognitive, perceptual, or emotional processes, the available studies investigating these oscillatory responses in children are limited [43,44,45]. A developmental trajectory observed in neuroimaging studies highlights changes in the connectivity between the amygdala and the ventromedial prefrontal cortex (vmPFC), a network essential for processing and updating emotional stimuli. This trajectory is characterized by an inverse pattern, transitioning from positive connectivity in childhood to negative connectivity during adolescence and continuing into adulthood [46].

In a study investigating different age groups, 10-year-old children showed bilaterally distributed delta responses to judging whether written word pairs matched their semantic, phonological, or orthographic features, whereas adults had higher left-lateralized delta responses. This difference was interpreted as the incomplete maturation of the left lateralization of the children’s linguistic network [41]. It is known from fMRI studies that by age three months, the infant cortex is already structured into several regions of functional importance for speech processing, for example, Broca’s area and anterior temporal regions [47], while the neural mechanisms underlying synchronization with the auditory envelope during speech listening are actively debated in the adult literature [48]. In a study comparing filtered visual evoked potentials of 3-year-old children and adults, children’s delta responses were similar to adults [49]. However, the post-stimulus latencies of their responses varied greatly. Furthermore, when perceiving ambiguous motion patterns, 10-year-old children had lower inter-trial coherence in their delta responses than adults [50].

Research indicates that in infants, cortical signals in the delta and theta frequency bands align with the rhythmic patterns observed in the speech envelope. Evidence suggests that in the first year of life, delta band tracking of the speech envelope is significantly more robust compared to theta band tracking, with the strongest delta tracking observed at four months. The power spectral density (PSD) of the delta signal produced in response to the stimulus maintains a consistent level from the age of 4 months, while the power in the theta band shows progressive development from 4 to 11 months [51]. It has been suggested that the delta power associated with the speech envelope could serve as a basis for the attentional and grouping mechanisms essential for the perceptual structuring of the speech signal, as evidenced by research conducted on adults [51,52].

Slow-frequency oscillations are fundamentally involved in the operation of widespread networks, whereas fast-frequency oscillations are primarily associated with the functioning of more localized networks [53]. Lower frequency bands, including delta and theta, might reflect the activity of interconnected neuronal groups and could play a role in facilitating information exchange across different regions of the brain [54,55]. In Nanova et al.’s (2011) investigation into event-related responses to auditory stimuli, an elevation in the delta, theta, and slow alpha power across all electrodes was noted in girls during the first 300 milliseconds following the stimulus. A deeper exploration of the data uncovered that in older girls, the observed alterations in delta frequency amplitude were underpinned by event-related phase coherence. The observation of gender-specific developmental differences in delta responses is considered additional evidence of the participation of extensive neural networks in auditory processing. The outcomes of the article reveal that the quickened functional maturation of processing networks is selective to particular frequencies and cannot solely be explained by a broad enhancement of neural connectivity [56]. Delta–beta coupling analyses are employed to indicate regulation capabilities in resting state recordings of infants, yet intra-individual fluctuations are observed within very short time spans. In interpretations made in this context, it is suggested that improvements in regulation abilities among children might be indicated by increased delta–beta coupling scores [57,58].

A review of the literature reveals that delta oscillations have been examined in contexts such as early language acquisition and stimulus processing, often in conjunction with slower frequencies like theta or low alpha. While delta oscillations suggest broad cortical activity, it is important to recognize that they do not solely capture cortical communication in early development. It is suggested that future research should explore the integration of delta oscillations with faster frequencies.

### 3.2. Theta Oscillations: Bridging Perception and Cognition in Early Years

The enhancement of theta rhythms in EEG has been linked to attention and anticipation processes between associative cortex areas and the hippocampal-frontal-parietal regions, leading to the identification of the ‘Theta-Response System” [59,60]. Additionally, the theta rhythm is recognized as a phylogenetically ancient mechanism crucial for neuronal organization within the brain, playing a pivotal role in learning [61,62,63]. From six months, infants start to direct their attention towards environmental stimuli. Köster et al. (2019) observed that visually entrained theta oscillations intensified in response to unexpected stimuli, indicating disrupted infant expectations. They utilized a novel experimental approach to manipulate oscillations in infants, offering fresh insights. Understanding the impact of stimuli on oscillations in children is vital for precise comprehension of brain dynamics [63].

Xie et al. (2018) indicated that sustained attention in infants is linked with theta (2–6 Hz) synchronization and alpha (6–9 Hz) desynchronization. During periods of prolonged attention, theta synchronization is evident across the frontal pole, temporal, and parietal electrodes, signifying enhanced allocation of attention [64]. Orekhova and Stroganova’s 1999 study, which examined the development of theta frequency in infants aged 8–11 months, discovered a theta frequency response in the frontal region of 8-month-old infants within the 3.6–6.0 Hz range across three distinct attentional states [65]. However, it was observed that while this response contributes to prolonged attention spans in the 9th and 10th months, the theta frequency response diminishes with age. This reduction in theta frequency response is believed to be a result of the decreasing need for activity to sustain attention as the brain matures and attentional capabilities improve [65,66]. In addition, the study examined the relationship between medial frontal theta power and inhibitory control in a large developmental sample and defined that non-phase locked medial frontal theta power plays a significant role in inhibitory control and task performance among children and adolescents [67].

Meyer et al. (2022) identified that infants displayed enhanced attentional engagement, as shown by increased theta power in the frontal area when they were presented with action demonstrations involving movements of varying amplitude, compared to demonstrations with consistent or high amplitude movements. Infants who exhibited increased theta power in response to the variable amplitude movements were more adept at successfully carrying out actions and engaging with novel objects relevant to the task at hand. This study supports the notion that infant-directed actions play a critical role in amplifying infants’ attentional capacities, learning processes, and exploratory activities [68].

Even though the resting state EEG studies have mentioned that the activation of theta with age is decreasing, there is a study which was conducted in 8-month-old babies where the working memory task creates a theta frequency response in a large part of the cortex [69,70]. In children, it is observed that the theta response is elicited in the medial frontal region, and the theta power decreases with age in the entire topography [71,72]. In addition, it is thought that the theta increase in the frontal regions in children occurs due to its relationship with executive functions [70,73,74].

In attention-demanding tasks, the increase in frontal theta oscillations helps individuals focus their attention on the target task while preventing distraction. Theta oscillations are associated with the processing of information and the activation of information relevant to the current task. Additionally, frontal theta activity establishes a connection between attention and memory, promoting better processing and retention of information [75]. As a result, bursts in frontal theta activity, which are facilitated by the activation of the protease calpain, increase myelination and connectivity [76]. In their 2018 study, Conejero et al. found that toddlers displayed a higher theta power at frontal electrode site when making errors compared to when they were correct. They propose that frontal theta activity plays a crucial role in modifying white matter fibers, potentially as a key factor in fostering the development of ideal structural links within the executive attention network [77]. In their 2021 research, Köster et al. explored the impact of prediction errors on brain oscillations in 9-month-old infants, finding heightened activity within the 4–5 Hz power range during unexpected events. The study demonstrated a significant rise in theta activity specifically associated with processing prediction errors in the infant brain. The authors theorize that besides encompassing all known information about theta oscillations, the theta rhythm might facilitate a computational strategy that condenses real-time events into a quicker neural timeframe. Furthermore, this could potentially enhance cognitive processes by allowing them to operate ahead of actual time and assist in incorporating new events into established networks [78].

The findings reveal that theta power increases in the frontomedial region when children are engaged in a task and increases further during the cognitive engagement. Theta power is also stronger over left fronto-temporal sites for language-related demands compared to action-related demands. The results suggest that theta oscillations in the frontomedial and anterior cingulate cortex reflect top-down control processes in 4-year-olds. Theta oscillations have the potential to serve as a neural marker of top-down control in early childhood and can provide insights into aspects of early development that are not directly reflected in overt behavior [79]. In the research involving infants exposed to Spanish and English syllables, Ortiz-Mantilla et al. proposed that an elevation in delta/theta power and phase coherence might support the auditory discrimination of syllabic content in 6-month-old infants [80].

As it is termed working memory, it depends on the persistent firing of neurons. There is converging evidence that this long term potention has an oscillatory character and that the frequencies involved are in the theta (4–8 Hz) and gamma (30–100 Hz) ranges [81,82]. In an animal study examining memory performance and theta oscillations, it was noted that theta responses were recorded during the encoding and retrieval phases, while gamma responses became more prevalent as the decision-making duration increased [35]. Further information about the necessity of the oscillations comes from the examination of the firing of individual cells while the short-term memory processes drive the information into long-term memory. In many brain regions, the neurons’ firing rate is very low, and cells do not fire during every cycle of the theta cycle. However, the firing is nevertheless organized by theta oscillations, as demonstrated by the fact that firing occurs selectively at certain phases of the theta cycle [83]. Superior performance on an infant working memory task has been associated with baseline-to-task increases in 6–9 Hz brain electrical activity at frontal sites during the stages of short-term encoding, storage, and retrieval [84]. Infants who successfully displayed ordered recall showed a pattern of increasing EEG from baseline to task at anterior temporal scalp locations, whereas infants showing no ordered recall displayed no changes in EEG from baseline to the task. The data in the study are the first to provide evidence to trace the continuous electrophysiological change in infant declarative memory processing throughout the creation of new event memories and long-term retrieval of events [85].

Although the medial prefrontal cortex (mPFC) is known as a neural generator of frontal theta oscillations, its potential maturational effects are not clearly understood. Moreover, mPFC develops significantly during early childhood [86]. There is an fMRI study which was conducted with 3-month-old infants. They have explained the hippocampal mechanism for the statistical learning paradigm and the activity of frontal sides during the task. Despite the hippocampus doubling anatomical volume across infancy, learning-related functional activity bore no relationship to age. They suggested that the hippocampus is recruited for statistical learning at the youngest ages in their sample. Outside of the hippocampus, they observed the involvement of the mPFC in statistical learning [87]. This is extremely surprising, given the dramatic changes in frontal lobe anatomy over development [88]. Consistent with recent research suggesting that the frontal cortex plays an important role in infant cognition [89,90]. In adults, mPFC strongly interacts with the hippocampus during memory formation, facilitating encoding based on related past experiences (i.e., schemas) to promote memory integration [91].

The study examined how proactive and reactive processing modes impact sensory and cognitive information processing in children aged 7–10 years. The findings indicated that the proactive processing mode was associated with increased theta activity before the stimulus and a reduction in the temporal synchronization of theta/alpha oscillations related to the event within the initial 300 ms following the stimulus. The outcomes suggest that holding internal task representations in working memory activates oscillatory networks, which in turn can influence the processing of incoming sensory data [92]. In research involving a large sample size of 239 children and adolescents, it was discovered that delta, theta, and alpha oscillations collectively contribute to the attentional engagement and focus necessary for working memory tasks. Within this synergistic relationship, slower frequencies, specifically delta and theta, play a crucial role in maintaining the active state of the working memory trace [93].

In a study investigating the impact of gender differences on working memory and auditory processing, it was observed that only the slower frequency bands (delta, theta, and slow alpha) showed an increase in phase-locking among girls, with no such increase noted in the faster (fast alpha) bands. It is thought that the rapid maturation of processing networks is selective for specific frequencies and cannot be attributed merely to a widespread strengthening of neural connections that would boost synchronization across all oscillatory networks [56]. Research investigating source memory identified elevated theta activity in 6–8-year-old children during a memory task, with no significant variation observed between conditions (fact and source recall). The increase in activity from baseline to task, recorded via frontal, temporal, and parietal electrodes, is speculated to indicate hippocampal cortical oscillations, which might signify episodic memory involvement [94].

Pre-verbal children have top-down mechanisms related to language encoding and attentional prediction of speech [95,96]. Another remarkable situation is the emergence of the theta response to emotional stimuli in infants [73,97]. A study examined the theta response of infants and children and compared them in three different attention conditions. The conclusion is the theta response increases in both infants and children in attention conditions. While theta frequency was observed at 3.6–5.6 Hz in the posterior temporal, occipital–parietal–temporal regions of infants under attention conditions. Theta frequencies in the frontal region of children are observed to be in the range of 4–7.6 Hz. One of the important results of the study is that the theta response, which occurs under attention conditions, increases at a greater topography in infants compared to children [73]. Cuevas et al. (2022) analyzed variations in EEG power across three frequency ranges (3–5 Hz, 6–9 Hz, 10–12 Hz) during a verbal recall activity in children aged two. The study highlighted that theta (3–5 Hz) and low alpha (6–9 Hz) rhythms provide significant insights into toddler memory processes. They found that as performance improved, the power values in these theta and alpha bands also increased, enabling the differentiation of toddlers according to their performance levels [98].

In a study focusing on 5-month-old infants observing changes in behavior and physiology in response to facial expressions, an increase in theta activity within the 4.8 Hz range was noted across most anterior and right posterior temporal regions of the scalp when infants were exposed to a blank face [99]. These findings suggest that infants are responsive to variations in adult facial expressions and indicate the possibility of distinct neurophysiological processes underpinning the infant’s gaze towards smiling versus neutral faces. Moreover, the presence of right temporal theta activity could reflect interactions between temporal cortical areas and the hippocampus. Given that hippocampal theta activity relies on cholinergic inputs from the nucleus of the solitary tract (the sensory center of the visceromotor component of the social engagement system), it may facilitate the cholinergic effects on the temporal cortex’s role in directing anticipatory attention towards the emotional content of faces [99,100,101]. In research examining speech perception through the integration of auditory and visual stimuli, the synchronization between theta phase and gamma amplitude was characterized as the interplay between top-down and bottom-up mechanisms in speech processing. The study found that the right hemisphere exhibits greater theta–gamma coupling under conditions where stimuli match, whereas the left hemisphere shows enhanced theta–gamma coupling when stimuli do not match. This distribution across hemispheres suggests a rapid global processing approach in the right hemisphere for congruent conditions and a more meticulous, slower processing method in the left hemisphere for dealing with incongruent situations [102].

The functional roles of theta oscillations have been elucidated in a variety of contexts, ranging from information processing to working memory capabilities, as detailed earlier. Table 1 comprehensively summarizes the technical specifications for all the articles reviewed in this study. The spatial distribution of event-related theta oscillations during childhood seems crucial for cognitive functions. Frontal theta activity is linked with attention processes and engaging in memory tasks, whereas temporal theta activity is associated with the processing of facial expressions and social engagement. Furthermore, with maturation, theta frequencies can be distinctly differentiated from those in the alpha range. For this reason, studying both theta and alpha frequency ranges concurrently in children is believed to be beneficial for gaining a deeper understanding.

### 3.3. Alpha Activity: Markers of Attention and Rest in Developing Minds

The alpha rhythm in early childhood functions as an alpha rhythm in adulthood but at a lower frequency. The 6–9 Hz band is suggested to be similar to the adult alpha rhythm for infants and toddlers [103]. Different frequency bands identified in infant EEG studies are slightly lower than those reported in adults but may support similar cognitive processes such as attention, memory, and emotion [54]. Alpha activity is increased in adults at rest, while decreased alpha is triggered during cognitively demanding tasks [104]. It is stated that alpha rhythms reflect activity in the thalamocortical feedback loop, as well as memory processes [29,105]. In a study using spectral analysis to examine longitudinal baseline data, 6–9 Hz was found to be the predominant frequency in most scalp electrode sites from infancy to 4 years of age. Therefore, the infant/toddler’s 6–9 Hz rhythm during silent wakefulness should be considered just like the adult’s 8–13 Hz alpha rhythm [103]. The findings from Yordanova et al.’s study suggest that the alpha response system is functionally active in children aged 6 to 11 years, although its development remains incomplete by age 11. The study indicates that the magnitude and phase-locking parameters may be associated with various functional aspects of the alpha response system. Consequently, while younger children can generate alpha responses during information processing, they do not engage this system as effectively as older children and adults [106]. Another study focused on alpha responses discusses that stimulus-locked alpha responses noted that when spontaneous alpha rhythms are absent, as seen in 3-year-old children, stimulus-locked alpha responses to visual stimuli are not recorded [107]. One of the studies explaining the complex nature of alpha oscillations mentions that alpha phase locking depends on age, topography and pre-stimulus alpha power in a way that is different from alpha amplitude. The ability to reorganize alpha activity after auditory stimulation and produce repeatable alpha patterns is emphasized in relation to brain development processes with age [108].

Alpha oscillations can be modulated both by internal rhythms and external stimuli, and studies on the mechanism of the relevant system continue. Considering the ability of interneurons specialized to work in the communication of certain cell groups to produce alpha oscillations, or the contribution of structures such as the thalamus to the production of relevant oscillations that are reflected in all brain regions, understanding the rhythm of the developing brain will also contribute significantly to interpreting the nature of the oscillations [109]. It is mentioned that low-frequency rhythms, such as alpha oscillations, provide synchronization characterized by a larger spatial volume [53,110,111]. As the time it takes for signals to travel between linked regions usually increases depending on the distance of spatial separation, the observed developmental rise in lower-frequency EEG patterns like mu and alpha might stem from improved signal speed across extended (anterior to posterior) white matter pathways. This enhancement could be due to the growth and development of axons and the myelination process, which may extend into early adulthood [21,112].

In an experiment involving maintenance and shifting of attention in 7–10-year-old children, researchers were able to describe the pattern of alpha oscillations modulated by attention. They explain that children tend to focus on the right side because the alpha oscillations in the left hemisphere are not strong enough to break the focus of attention. Considering that the ventral network, controlled by structures around the right temporal parietal junction (TPJ), is required for extrinsically directed attention switches, they explain why this effect applies only to the right side. It is argued that lateralized alpha modulation can be used as a tool to examine attention processes in groups such as children with attention disorders since alpha modulation is already present at these ages [113]. At the source level, seed-based connectivity analyses show that sustained attention induces a reduction in the alpha frequency band (localized within dorsal attention and default mode networks) [114].

Resting state activity continues to change during childhood and adolescence, during which the amplitude of delta and theta frequency activities decreases, while faster frequency band activities (alpha, beta, and gamma) increase [115,116]. This dynamic is a sign of central nervous system (CSN) maturation [69,117]. Alpha oscillations with a 6–9 Hz frequency are related to visual attention, emotional expression, working memory, and inhibitory control in infants [85,118]. When time–frequency (TF) analyses were applied to EEG data collected from 50 infants at different times (8 months and four years old) using a working memory task, the working memory process was found to be associated with increased alpha power (6–9 Hz) at an average of all electrodes at eight months, and for medial anterior electrodes, it was determined to occur at four years [70]. This more focused activity was interpreted as an increased functional specialization through development [119,120]. In the previous study, we discussed the increase in theta and alpha power and phase locking in the parietal–occipital regions to the objects remembered of 6–7-year-old children’s visual and auditory memory tasks [121]. Moreover, while beta and low-frequency components work together for encoding, it is mentioned that alpha oscillations sharpen this process. In a study with children involving memory tasks, reaction time is used as a marker of behavioral responses and has the most consistent relationship with the alpha (8.5–10 Hz) frequency range [93]. Alpha phase cycles contribute to the simultaneous functioning of brain regions by creating a time window for excitation or inhibition [122,123], which is thought to be important in attention and learning functions. In a study comparing sensory-evoked potentials in children and adults, auditory-evoked potentials were detected and considered to be a superposition of rhythms in the delta and theta ranges. It is also mentioned that 3-year-old children do not produce an alpha resonance response to sensory stimuli [124].

Upon reviewing all articles listed in Table 1 within the scope of this study, spectral analysis of the alpha frequency range emerges as a crucial method for detecting and interpreting shifts in frequency during neurodevelopmental processes. The phase and power data concerning the event are believed to be instrumental in delineating the link between functional development and brain oscillations in children. Given that infants in their early life stages need to interpret environmental signals critical for survival and managing physiological needs, the gradual transition from slower oscillations indicative of autonomic regulation to faster oscillations that underlie higher cognitive abilities is natural. It is thought that as infants develop skills, the evolution in functions and corresponding brain oscillations occur concurrently.

### 3.4. Mu Rhythms: Mirroring Movement and Social Cognition

Over the years following its initial identification, the mu rhythm in adults has been defined by three primary characteristics: specific regional brain distribution, particular frequency range, and a reliance on behavioral context for its activation. In practical terms, the mu rhythm is notably recognized for its reduced synchronization during deliberate muscle activity [21,125]. Differing from the posterior alpha rhythm, the mu rhythm remains relatively stable and is not significantly altered by shifts in environmental lighting conditions [126]. Due to the significant role of this rhythm, motor execution has emerged as the definitive functional criterion for defining the mu rhythm [21].

The characterization of mu oscillations acknowledges a distinction between the upper (10–13 Hz) and lower (8–10 Hz) mu band frequencies. Pfurtscheller et al. (2000) advocate that these bands exhibit distinct functional and spatial characteristics, where the upper band is associated with a more localized, movement-specific ERD pattern, unlike the lower band, which displays a widespread and more generalized ERD pattern [21,127]. The link between movement perception and execution in infants has been explored through EEG research. Substantial event-related desynchronization was noted in the central regions when infants engaged in intentional activities, and a similar desynchronization occurred in these regions while observing the same actions. Task-related studies suggest that the central rhythm in infants, occurring at 6–9 Hz, differs from the alpha range rhythmic activity observed in other areas of the scalp. It is mentioned that this research supports the notion that the infant rhythm exhibits functional similarities with the adult mu rhythm [128].

Motor related mu supression develops early in children. The power of mu rhythm (8–14 Hz) decreased significantly during movement, and it returned to baseline after movement for both children in 7-year-olds [129]. Morover, the reduced mu power is shown in infants with task-dependent activity (congruent to incongruent stimuli). Central electrode sides (C3–C4) might be evaluated as primary channels of interest in studying mu rhythms (6–9 Hz). It is suggested that occipital channels are unavailable because of the widespread posterior alpha effect [130]. The parietal cortex is recognized for supporting the exact kind of visuomotor integration necessary for performing reaching and grasping tasks, which is frequently used in EEG investigations of the mu rhythm [131,132]. It has also been demonstrated that mu suppression correlates with changes in the BOLD (Blood Oxygen Level-Dependent) signal within the premotor and parietal regions [133]. Central–parietal desynchronization, which is thought of as representitive of the neural mirroring, was found in a study using a paradigm based on active and observational learning in children. They found 3- to 6-year-old children exhibit neural mirroring within the mu ryhthm [134]. Given that the mu rhythm responds to both the execution and observation of motor actions, researchers have advocated for the utilization of mu rhythm modulations as an effective instrument for investigating the human mirror neuron system [135].

The mu rhythm exhibits a comb-like appearance, indicating it consists of two primary frequency components that have a nearly harmonic relationship, specifically the alpha (10 Hz) and beta (20 Hz) frequency bands. These two components of the mu rhythm are subsequently referred to as “mu alpha” and “mu beta,” based on their spectral properties. The two frequency components of the mu rhythm can be influenced by various stimuli or tasks, such as somatosensory stimulations, imagined movements, observed movements, the redirection of spatial attention, and the anticipation of focused stimuli [21,136]. Greater occipital alpha (8–10 Hz) ERD is found during the perception of the active training task in children. This suggests that occipital alpha may be facilitating the processing of attended stimuli while suppressing attention to irrelevant information [134]. ERD of the mu rhythm (including both mu alpha and mu beta) indicates sensorimotor network activation, while ERS or the rebound observed after movement is considered to represent processes of top-down inhibitory control [137]. Morover, it is found that anticipatory ERD of the mu rhythm is related to executive function skills, whereas it is not associated with the accuracy of children’s behavioral responses to tactile stimulation [137].

ERD and ERS can be altered by different parameters of movement, including its strength, duration, frequency, and complexity [138]. Observing a real action situation led to greater power desynchronization in the μ range in the medial frontal and medial parietal loci compared to observing a fake action situation. Children with higher levels of speech comprehension showed greater EEG power desynchronization in the μ range when observing a real action in the frontal and parietal loci of the left hemisphere [139]. The results suggested that children with higher levels of speech comprehension have higher activation of the mirror neuron system, which has a key role in forming and perceiving speech [139,140].

From the moment of birth, children start to develop motor skills. The organization of attention and limb movement is crucial for mastering a motor skill, and analyzing the event-related mu rhythm in children offers valuable insights into the construction of movements. Within this framework, the mu alpha rhythm contributes to cognitive reserve, while the mu beta rhythm delivers significant details about the proficiency level of the executed movement. While language development primarily involves slower frequencies, the spatial proximity of the mu rhythm to language regions and the required coordination of jaw muscles for speech exemplify the mu rhythm’s significance for understanding brain dynamics.

### 3.5. Beta Oscillations: The Rhythms of Active Thinking and Problem-Solving

The beta frequency band comprises small-amplitude fast oscillations, and these characteristics, along with short time spectra, may facilitate identification. It exhibits activation patterns during action observation in adults, and similar changes in brain activity are observed during developmental processes. In research involving 3-year-old participants, variations in beta-range power were associated with time-locked motor responses to a partner’s actions, whereas changes in mu power suggested a broader engagement of the motor system during a cooperative task. Even in early childhood, the way the motor system integrates the actions of others varies depending on whether children are involved in a joint action. This context-dependent activation of the motor system may have significant implications for the development of successful cooperative behaviors in joint activities. Furthermore, a more pronounced time-locked increase in beta power correlated with fewer mistakes made by children in acting jointly [141]. Köster et al. (2000) showed an increase in motor cortex activity within the 7–10 Hz range during the observation of actions, which anticipated the imitation of actions in 20-month-old infants. Infants at ten months of age, who were not yet consistently imitating the actions of others, exhibited a neural activity pattern remarkably similar during the observation of actions [142]. Liao et al. (2015) identified the sources of the mu rhythm in toddlers as being located in the somatomotor cortex, likely in proximity to the hand/arm homunculus within the central sulcus. This finding underscores the recent progress in signal analysis techniques, which have significantly enhanced the precision of EEG source localization [143].

In a study focused on developmental training investigation of the sensorimotor beta rhythm, they found a significant central–paeriatal ERD during action execution but not action observation. They have also added that there are no training effects on the beta rhythm during action perception. The decreased beta ERD has been associated with conceptual knowledge of actions [134]. Moreover, whereas conceptual training decreases the beta ERD, motor training increases activity [144]. It was argued that the beta rhythm might be more reactive to the observation of complex actions that need relatively long-term training, and numerous factors can influence beta ERD; thus, further research is required to identify the specific contexts and variables that modulate this activity [134]. With another perspective, beta power (15–30 Hz) has different dynamics between children and adults. The movement induce beta ERD in both, but post-movement beta power rebound (PMBR) is observed only in adults. PMBR might be associated with a more prolonged motor development process [129].

In adults, the power of somatomotor mu rhythms and their beta harmonics is reduced during the execution of intentional actions or while observing the actions of others [145]. Liao et al. (2005) observed a reduction in beta power in the left hemisphere during action execution, suggesting this phenomenon as a result of hemispheric lateralization and the design of unilateral tasks. However, they also emphasized the need for further studies to explore the connections between mu and beta rhythms in upcoming research [143].

A study evaluating the family difference between family-raised children and orphans’ performance during the speech perception task showed that during direct speech perception, beta and gamma oscillations were synchronized. However, in the institutionalized group, the synchronization of these rhythms was less pronounced. The presence of a pronounced beta rhythm in EEG data is interpreted as indicating an activated state of both cortical and cortico-subcortical neuronal networks [146]. Vrobel et al. (2000) suggested that beta-frequency oscillations in cortico-thalamic pathways are associated with a reduced activation threshold of thalamic relay neurons. This reduction improves the specificity of information flow to targeted neocortical areas during tasks that require attention [147]. Angel and Fries (2010) put forward a theory regarding the significance of the mechanisms behind high-frequency EEG rhythms (beta and gamma) production. They suggested that during motor and cognitive tasks, the beta rhythm facilitates the maintenance of the current motor state and aids in the sustained and routine execution of cognitive activities [148].

A reduction in beta power, which previous studies have linked to efficient syntactic processing, was observed [149,150]. Research exploring the developmental evolution of neural oscillations in auditory sentence comprehension identified developmental distinctions through neural oscillations and ERP dynamics. Findings revealed that children display a distinct pattern of involvement, showing an absence of the beta power reduction in response to grammatical errors observed in adults [146]. It was demonstrated in another study investigating the processing of auditory stimuli that time–frequency analyses of the EEG data that are collected from infants’ data indicate that the stimulus evoked 1/f pattern neural synchrony like adults. The stimulus onset shows synchronous bursts of theta, beta, and gamma power in the brain’s right and left auditory regions. This article is on the concept of technical details and approach for EEG data, and they have suggested the creation of a model to understand the integrity of early auditory processing mechanisms for future studies [30].

Children encounter numerous novel experiences and develop various skills from an early age. As previously detailed, this involves activities like focusing attention, processing data, or monitoring their surroundings. Here, it becomes apparent that the beta rhythm plays a crucial role among the rapid frequencies that escalate as children age. While predominantly linked to motor activities in adults, beta oscillations in children indicate functional capabilities across various domains, including language development, movement observation, and the interpretation of social signals.

### 3.6. Gamma Band: High-Frequency Insights into Advanced Cognitive Integration

The production of the gamma rhythm is associated with the functioning of pacemaker neurons located in the specific and intralaminar nuclei of the thalamus, where these cells produce bursts of spikes at a frequency of approximately 40 Hz. This activity enhances and synchronizes neuronal activity in select cortical areas, providing the neurophysiological foundation for the efficient integration of incoming information and consciousness [146,151]. When a person detects a significant external stimulus demanding extra cognitive work, gamma oscillations are among the rhythms that become more pronounced. The synchronization of EEG oscillations in the gamma-band frequency can quickly connect cell ensembles spread across different locations, thereby offering the required speed for cognitive processing [148]. Gamma oscillations have been associated with various sensory and higher cognitive abilities, including feature binding, attentional processes, and working memory capacity [152,153,154]. Furthermore, it has been proposed that these oscillations be generated by several cellular and synaptic mechanisms [110]. As a corollary of this, these oscillations are sensitive to the maturational changes in the brain. Therefore, understanding the characteristics of these oscillations across developmental stages will provide further information about the development of sensory and cognitive abilities in humans. However, when compared to studies on slower frequencies, the number of studies on gamma event/task-related oscillations in children is limited.

Consistent with the previous findings on the effects of social gaze on infants’ EEG activity, 4-month-old infants had larger gamma band responses to faces with direct gaze compared to faces with averted gaze, pointing to the importance of this ability in social interactions even in the earlier stages of the development [155,156]. Furthermore, object permanence ability in infants has been shown to be developing earlier than previously thought. Specifically, 6–8-month-old infants had higher gamma band activity in response to maintaining visual representations of two occluded objects versus one [157]. Also, in order to eliminate the potential effects of object memory on this greater gamma response over the right temporal region, researchers compared different types of object disappearance (occlusion vs. disintegration) and concluded that the detected increase was linked with the continued presence of the object, not the object memory [158,159]. On the other hand, the occlusion of face stimuli did not elicit this response, implying the presence of distinct mechanisms underlying these processes. In addition to the processes mentioned above, violating infants’ expectations regarding completing a goal-directed chain of events elicited higher power in gamma responses in the left frontal area [160,161]. Also, infants demonstrated a preference for their native language as early as six months, which was evident by the increase in their induced gamma band responses [80].

Similar to adults, children demonstrated increased gamma band power in response to moving grating stimuli as well, which was predominantly observed over the occipital area [162]. Also, the peak frequency of their gamma oscillations was increased in response to the increasing velocity of the moving gratings. Moreover, compared to institutionalized orphans, children who their families raise had higher synchronization in the gamma frequency band in response to direct speech [146]. This difference was linked to developmental delays in language and cognitive abilities of the institutionalized children. Furthermore, target auditory stimuli elicited larger gamma band responses (within 0–120 ms) with stronger phase-locking in children, which was interpreted as being related to earlier attentional mechanisms [163].

## 4. Concluding Remarks

This review has provided a detailed examination of the developmental trajectories of brain oscillations from infancy through childhood, highlighting the significance of delta, theta, alpha, beta, mu, and gamma rhythms in various cognitive and sensory processes. Each oscillation band presents unique characteristics and developmental patterns that contribute to the overall understanding of brain maturation.

Delta oscillations are crucial in early development, particularly in language acquisition and cognitive processing. Their decrease with age signifies the maturation of cortical structures. Theta rhythms bridge perception and cognition, with their role in attention and memory becoming more specialized as children grow. Alpha activity, reflective of thalamocortical interactions, shows increased complexity and is essential for inhibitory control and memory. Beta rhythms are vital for active thinking and problem-solving, showing distinct patterns during motor and cognitive tasks. Mu rhythms, which reflect motor control and social cognition, highlight the importance of the mirror neuron system in learning and social interactions. Gamma oscillations are associated with high-level cognitive functions such as attention, memory, and perceptual binding, showing significant developmental increases in response to sensory and cognitive stimuli. Understanding these oscillatory patterns and their developmental trajectories provides a foundation for further research into the neurobiological underpinnings of cognitive and sensory development. This knowledge is essential for identifying biomarkers for cognitive and developmental disorders, offering potential for early diagnosis and intervention strategies. Future studies should focus on longitudinal analyses to capture the dynamic changes in brain oscillations and explore their implications for clinical practice.

The integration of advanced neuroimaging techniques and computational models will enhance our ability to interpret the complex interactions between different oscillation bands. Additionally, examining the impact of environmental factors, such as early education and social experiences, on the development of brain oscillations will provide valuable insights into optimizing developmental outcomes. In summary, the intricate patterns of brain oscillations from infancy to childhood reflect the profound changes occurring in the developing brain. These oscillations serve as vital indicators of neural maturation and cognitive development, offering a window into the functional architecture of the brain. By continuing to unravel the complexities of brain oscillations, we can pave the way for innovative approaches to support healthy development and address neurodevelopmental challenges.

## Figures and Tables

**Table 1:** Summary of key articles reviewed.

	Development Stages and Focused Oscillations	Modality and Paradigms	Inference
Musacchia et al. (2015)	211 infant recording sessions with 98.6% of data retention 57 participants Theta, beta, and gamma oscillations	The auditory stimuli in free-field. Stimuli are presented in two blocks that include a fundamental frequency of either 800 or 1200 Hz and 15 harmonics (6 dB roll-off per octave).	Time-frequency analyses of the EEG data that are collected from infants’ data show that stimulus evoked 1/f pattern neural synchrony like adults. The stimulus onset shows synchronous bursts of theta, beta, and gamma power in both the right and left auditory regions of the brain. Suggestion about the creation of a model to understand the integrity of early auditory processing mechanisms.
Güntekin et al. (2023)	40 participants (children and young adults with rates of 1 in 2) Alpha oscillations	The day-night stroop task. Event-related spectral perturbation (ERSP) and event-related phase-locking analyses were performed.	Different alpha dynamics in children. Shifting of alpha power from posterior to anterior with age. The posterior sides of the brain are involved in the inhibitory processes. Increased alpha power in early time windows, decreased event-related desynchronization (ERD) in later time windows.
Gómez et al. (2023)	239 participants (6–29 years) Delta, theta, and alpha oscillations	The Delayed-Match-to-Sample Test. ERSP in the range of 1–25 Hz.	Delta, theta (7–8 Hz) and alpha-2 (8.5–10 Hz) oscillations work for attentional engagement and focusing for working memory. Beta and low-frequency components work together for encoding, while alpha sharpens this process. Low-frequency component works to keep the WM trace active. Anticipatory attention is characterized by an increase in alpha and beta in late adolescents and adults. Behavioral responses which are represented with reaction time have the most consistent relationship with alpha-2.
Wang et al. (2014)	17 children (mean age 10 years, 5 months) Theta and gamma oscillations	Speech perception paradigm consists of the match and mismatch conditions with auditory and visual stimulus. Theta–gamma coupling analysis.	The coupling of theta phase and gamma amplitude demonstrates the interaction of top-down and bottom-up processes in speech perception. Right hemisphere has higher theta–gamma coupling in the match conditions, and the left hemisphere has stronger theta–gamma coupling in the mismatch conditions. This hemispheric pattern is interpreted as a fast global processing strategy and a slow detailed strategy to adapt to congruent and incongruent conditions.
Ortiz-Mantilla et al. (2013)	28 infants (6-month-olds) Theta and gamma band	English and Spanish syllable contrasts. Single-trial time frequency analysis. Temporal spectral evaluation (TSE). Intertrial phase locking (ITPL).	Increased delta/theta power and phase alignment could subserve auditory discrimination of syllabic information in 6-month-old infants. Preferential processing of specific native features are represented by frontal gamma power. Six-month-old infants can distinguish phoneme information across languages.
Maróti et al. (2019)	22 children (mean age 6.5 years) Beta and gamma frequency range	Auditory stimulus includes the tones at the beta and gamma range in isochronous sequences. Steady-state evoked potentials.	Neural responses which are related with the entrainment associated with the different frequency oscillations do not develop at the same level.
Musacchia et al. (2017)	49 infants A longitudinal study at the fourth and seventh months Theta and gamma oscillations	Active and passive auditory exposure. Go/no-go task. Passive oddball paradigm. Three different types of paired acoustic stimuli. TSE and ITPL.	A representation of maturation from 4 to 7 months, which is increased spead of event-related source response and decreased theta phase stability. Differentiation of theta and gamma oscillatory dynamics between active and passive auditory exposure protocols. Acoustic experience appears to promote more mature oscillatory and lateralized patterns for the auditory system.
Colomer et al. (2023)	17 participants (9-month-olds) Theta and beta	Action observation task with familiar and novel conditions. Action execution task to engage the infants actively. Time–frequency analyses (4–20 Hz). Inter-Site Phase Clustering (ISPC) to perform phase-based connectivity analysis.	Infants’ brain has greater ISPC with age. It is suggested that the global connectivity at the whole brain might be related to maturation. Greater visual–motor coherence is shown in infants with better motor skills. That could have downstream consequences on how infants encode and learn others’ actions.
Hao et al. (2020)	16 children (mean age 7.7) and 13 adults Mu and beta rhythms	Motor-related experiment. Event-related spectral pertubations (ERSPs). Inter-trial coherence (ITC).	The power of mu rhythm (8–14 Hz) decreases significantly during movement, and it returned to baseline after movement for both children and adults. Motor related mu supression develops early in children. Beta power (15–30 Hz) has different dynamics between children and adults. Movement induces beta ERD in both, but post-movement beta power rebound (PMBR) is observed only in adults. PMBR might be associated with a more prolonged motor development process.
Adam et al. (2020)	87 children (52 preschool and 35 school children) Theta frequency range	Heart and flower task to evaluate cognitive control process. Behavioral results (response time and accuracy). Time–frequency analysis. Midfrontal theta peak latency.	Midfrontal theta power is interpreted as a generic indicator of cognitive control process that supports both inhibitory and flexibility functions. MFT might be accepted as a general neural mechanism to coordinate cognitive process across the development.
Cantiani et al. (2022)	A longitudinal study at seventh and ninth months 25 infants (mean age 8 months)	Three different rhythmic stimuli (African, Tabla, and speech). Steady-state evoked potentials (SS-EPs).	Low frequency cortical tracking of the stimulus envelope is already measurable at 4 months of age. There are no hemispheric differences in the magnitude of SS-EPs. There is left-lateralized SS-EPs at higher frequencies for speech stimulus. Infants can entrain like adults, but there is difference between frequency ranges for same stimulus.
Noordt et al. (2022)	432 participants (224 females, 208 males) Theta	Go/no-go task. Raven’s progressive matrices to measure reasoning ability. Threshold free cluster enhancement and permutation testing to analyze spatial and temporal dynamics of theta power. Phase evaluation. Latent class analysis.	Greater non-phase locked theta power during response inhibition is associated with more efficient response control. Developmental changes in frontally mediated inhibitory control processes are represented by a greater non-phase locked theta power and a shift to less phase locked activity.
Antognini et al. (2019)	20 toddlers (18 months), 27 toddlers (24 months) Mu	Three types of stimuli-related actions (auditory, visual and play material).	The lateralization of mu suppression towards the left hemisphere in response to action verbs in terms of similarities with language processing. Very strong occipital alpha suppression for action observation differs between age groups, whereas the lateralization of central activity is towards right hemisphere. Although the neural processing of action verbs and pseudoverbs is related to the left central cluster, the sensorimotor activity is shown just for action verbs.
Köster et al. (2021)	36 infants (9-month-olds) Theta	The violation of expectation paradigm to evaluate neural proccesing of prediction error. Time–frequency analysis and event-related potentails	All scalp electrodes has increased activity in 4–5 Hz power in unexpected events. Theta substantially increased for the processing of prediction errors in the infant brain. Theta rhythms are located in the parietal regions in infants. Theta serves as a facilitation mehanism to integration of new information into existing networks.
Patzwald et al. (2020)	38 infants (18-month-olds) Mu	Action observation with verbal cues. Mid-latency ERP 300–800 ms. Central mu power (6–9 Hz).	Reduced mu power is shown for congruent to incongruent stimuli. Central electrode sides (C3–C4) might evaluate as a primary channel of interest to study mu rhythm (6–9 Hz). Occipital channels are unavailable because of the widespread posterior alpha effect.
Bryant et al. (2019)	21 children (mean age 4.89) Mu and beta	Two tool sets and plastic aquatic animal toys. Active and observational training sessions to handle the toys. Spectral analysis of signals. Time-locked ERD.	Central–parietal desynchronization during the action observation and execution represents neural mirroring, and 3- to 6-year-old children exhibit neural mirroring within mu ryhthm. Greater occipital alpha (8–10 Hz) ERD is found during perception of the active training task. This suggests that occipital alpha may be facilitating the processing of attended stimuli while suppressing attention to irrelevant information.
Angelini et al. (2023)	17 infants (9-month-olds) Theta (4–5 Hz) and alpha (6–8 Hz)	Face-to-face live paradigm with a triadic social interaction. Time–frequency analysis.	Theta power increases in response to an unexpected event that is interpreted as a marker of information prediction about social behaviors. Higher alpha power is detected in infants in relation to the inhibition of interfering stimuli needed to sustain internally controlled attention. The live paradigm favors the expectation of a usual event that could have an enhanced effect on alpha power.
Ramos-Escobar et al. (2021)	60 children (mean age: 9 years and 5 months) Syllable and word frequencies in ROIs	Audio-visual artificial language streams. Frequency tagging analysis of spectral power. Weighted Phase-Lag Index.	Frontal and parieto-occipital ROIs at target frequencies are detected with long-distance EEG phase synchronization. Word segmentation and meaning mapping might be modulated by attentional mechanisms to multimodal word learning.
Weiss et al. (2018)	80 children (6 to 8 years old) Mu and alpha	Tactile stimulus. Flanker task. ERSP.	Anticipatory ERD of the mu rhythm is related to executive function skills, whereas it is not associated with the accuracy of children’s behavioral responses to tactile stimulation.
Mikhailova et al. (2021)	36 children (17–41 months) Mu	Observation of actions (fake, real, and performing the action). Bayley test for speech comprehension. Time–frequency analysis.	Observing a real action situation led to greater power desynchronization in the μ range in the medial frontal and medial parietal loci compared to observing a fake action situation. Children with higher levels of speech comprehension showed greater EEG power desynchronization in the μ range when observing a real action in the frontal and parietal loci of the left hemisphere.
Cantiani et al. (2019)	56 participants Typically developing (15 F/17 M) Age: 6 months and 20 months of age (longitudinal) High familial risk for Language and Learning Impairment (LLI) (11 F/13 M) Age: 6 months and 20 months of age (longitudinal) Delta/theta (2–12 Hz) Gamma (30–80 Hz)	Non-speech double oddball paradigm. Cluster permutation testing: Spectral power (TSE) Phase coherence (ITPL). Tests: BSID-III Language Development Survey (LDS) Communicative Development Inventories (CDIs).	Higher bilateral theta activation in auditory cortices in response to deviant stimuli in both groups Infants at risk for LLI: reduced gamma activity in left auditory cortex and higher gamma activity in right auditory cortex
Phelps et al. (2022)	48 participants 24 bilinguals (8 F/16 M) Age: (M = 9.3 years, SD = 1.83) 24 monolinguals (11 F/13 M) Age: (M = 9.6 years, SD = 1.48) Low-frequency neural oscillations	Dichotic listening task. Multivariate temporal response function (mTRF). Speech envelopes.	Different EEG patterns in monolingual and bilingual children during encoding Weaker differentiation of linguistic distractors in bilingual children
Köster et al. (2020)	42 participants (26 F/16 M) Age: 10-month-olds 36 participants (15 F/21 M) Age: 20-month-olds 2–14 Hz	Action demonstration videos and generalization test, followed by imitation test. Spectral perturbation analysis.	20-month-old infants: increased 7–10 Hz activity at C3-C4 during action observation; desynchronization in 3–6 Hz peaked at posterior electrodes (P3, P4, Pz, P7, P8, O1, O2)
Ossmy et al. (2021)	22 participants (11 F/11 M) Preschoolers Age: 3.09 to 5.49 years 22 participants (14 F/8 M) Adults Age: 19.37 to 26.40 years 6–9 Hz 8–13 Hz (Mu)	Videos of efficient and inefficient displays of tool use (multi-step actions). Eye-tracking, pupil dilation, EEG, and machine learning. Nonparametric cluster analysis. Event-related spectral perturbation (ERSP).	Increased event-related desynchronization in the mu frequency band at sensorimotor regions after action observation. Differential EEG responses to efficient versus inefficient initial grips observed only in adults.
Anderson et al. (2021)	55 participants (31 F/24 M) Age: 6–12 months Alpha (6–9 Hz)	EEG data: baseline (1–2 min), 90 s of mother/infant play Behavioral data: parent/infant play, infant temperament. Fast Fourier Transform (FFT). Tests: The Infant Behavior Questionnaire (IBQ-R).	Significant positive correlation between baseline frontal alpha asymmetry (FAA) and object exploration Only the cuddliness subscale (in IBQ-R) was associated with a leftward shift in FAA.
Georgieva et al. (2020)	14 participants (8 F/6 M) Age: (M = 338.85 days) Delta (1–3 Hz) Theta (3–6 Hz) Beta (∼15 Hz)	EEG data: resting state, spontaneous motion. Fast Fourier Transform (FFT). Cluster-Based Permutation Test.	Movement EEG scalp topology was similar to resting state EEG: high delta/theta power at posterior regions and high beta power at orbitofrontal regions. Upper limb movements characterized by increased beta power and generated more widespread artifacts. Decreased theta and alpha power at central in all motion types.
Ortiz-Mantilla et al. (2022)	100 participants (47 F/53 M) Age: 4 months (longitudinal visits at 7, 9, 12, and 18 months) P1 Theta (frequency clusters in 2–6 Hz range)	Auditory go/no-go task (target stimulus is paired with a reward video). Intertrial Phase Synchrony.	Lower theta phase synchrony at 7 months is linked to better expressive language at 12 and 18 months and better receptive language at 9 months.
Buzzell et al. (2020)	136 participants 68 receiving care as usual (CAUG) (35 F/33 M) 68 receiving foster care (FCG) (34 F/34 M) Age: (M age = 21.6 months) Mediofrontal theta (4–8 Hz) oscillations	Go/no-go task. Behavioral assessment of risk for psychopathology (HBQ). Theta power was calculated as the total power measure weighted by the average power time-frequency PCA loadings.	Higher error-related mediofrontal theta power in FCG compared to CAUG.
Rayson et al. (2019)	23 participants (10 F/13 M, Age: 6.5 months (M = 200.91 days, SD = 5.86) 24 infants (13 F/11 M), Age: 9.5 months (M = 292.92 days, SD = 7.88) Alpha	Preferential looking stimuli. Gaze following stimuli. Event-related spectral perturbation analysis.	At both ages, alpha ERD was higher in the congruent condition than the incongruent condition. The higher the alpha ERD in the congruent condition, the more preference toward congruent stimuli (9.5-month-old infants).
Haartsen et al. (2020)	73 participants (38 F/35 M, Age: (M = 302 days, SD = 13) EEG data for the second session consisted of 64 infants (9 drop-outs) Alpha oscillations Graph theory metrics (normalized weighted clustering coefficient, normalized weighted path length, and small-worldness index)	Presentation of naturalistic dynamic videos and moving toys (60 s each)—2 sessions with one-week interval. Phase lag index (PLI). Debiased weighted PLI (dbWPLI) measured using Fourier coefficients.	Intra-class correlations (ICCs) for whole brain connectivity were higher than ICCs for the other graph metrics.
Hoehl et al. (2014)	24 participants (9 F/15 M) Age: 8 months and 29 days Alpha oscillations	EEG recording during eye-contact and no eye-contact conditions. Event-related spectral perturbation.	Infants responded with alpha desynchronization to eye-contact condition. In no eye-contact condition no alpha synchronization or desynchronization effect was observed.
Conejero et al. (2018)	66 participants 52 toddlers (26 F/26 M) Age: (M = 16.75 months; SD = 0.67) 14 adults (13 F/1 M) Age: (M = 21.93; SD = 2.34) Error-related negativity (ERN) Theta (4–7 Hz)	27 different animal head/body combinations (conceptual error condition). ERP. Time–frequency (power).	Higher fronto-central negativity for incorrect trials compared to correct trials in all participants. Significantly greater increase in theta power in the error condition compared to the correct condition in children of highly educated parents. SES significantly contributed to the amplitude of the ERN.
Jones et al. (2015)	168 participants 88 infants (39 F/49 M) Age: 6 months 80 infants (40 F/40 M) Age: 12-month Theta (3 to 6 Hz) Alpha (6 to 9 Hz)	Two movies of 1-min duration: naturalistic social and non-social FFT.	Socially selective theta responses showed increased power and topographical extent in both Live Action and Movie formats between 6 and 12 months. Theta power has the potential to be a more sensitive measure of social brain development in the first year of life.
Thorpe et al. (2016)	20 adults (11 F/9 M) Age: 18–21 years old, 47 participants (21 F/26 M, 9 drop-outs) Age: 45–68 months 50 participants (3 drop-outs) Age: 12 months Lower alpha band (8–10 Hz) Upper alpha band (10–12 Hz) Upper mu band (10–13 Hz) Lower mu band (8–10 Hz)	Grasp execution task. Power spectral density (PSD). Event-related desynchronization.	Both alpha bands’ topographies peak over occipital–parietal electrodes in all subjects. Strong peak over frontal channels in both alpha bands in adults; no evidence of such a frontal peak in younger subject groups.
Friedrich et al. (2017)	107 participants (47 F/60 M) Age: 6–8 months 12–15 Hz	Learning phase and memory test (primed and unprimed word pairs were tested) ERP, FFT (late negativity, N400, spectral power of sleep spindle activity).	Evidence that infants as young as 6 months old can form semantic categories in long-term memory and associate them with word forms. The formation of perceptually based categories occur in the earlier stages of the sleep (NREM sleep stage 2).
Cuevas et al. (2011)	20 healthy, full-term infants (longitudinally seen from 5 months to 10 months of age) 6–9 Hz	EEG and ECG recordings: Baseline A-not-B task EEG power Coherence.	Baseline: (a) Power increased with age in all electrode sites except for the frontal pole and lateral frontal; (b) coherence increased between frontal pole/medial frontal, medial frontal/lateral frontal, medial frontal/medial parietal, and medial frontal/occipital; (c) heart rate decreased with age. Task: (a) evidence for working memory processing related increase in EEG power in 5-month-old infants; (b) only 5–7-month-old infants showed more localized task-related changes in EEG power in medial parietal region; (c) HR showed no task related changes.
Meyer et al. (2011)	7 participants (2 F/5 M) Age: 3-year-olds 17–21 Hz (beta)	Joint button-pressing game with three conditions: (1) joint action, (2) joint action observation, (3) individual action. Time–frequency representations. Discrete Fourier Transform (DFT)	Higher decrease in beta power in children during joint action observation when they were observing their own joint action partner. Enhanced motor system activity was observed at C3 electrode and was associated with fewer errors during task.
Swingler et al. (2017)	388 participants (199 F/189 M) Age: at 5 months and 10 months of age (longitudinal) 6–9 Hz	EEG Data: Baseline Visual attention tasks at 5- and 10-month follow-ups. Behavioral data: Attention (glove puppet presentation). Maternal behavior (child-mother interaction). Medial frontal EEG power (F3/F4) DFT—natural logarithmic normalization.	Increased EEG power difference at 10 months at the right frontal medial region was linked with better task performance. Higher positivity during mother–infant interactions promote better attentional engagement.
Cuevas et al. (2012)	122 participants (62 F/60 M) Age: 23 months–28 months (longitudinal) Theta (3–5 Hz) Alpha (6–9 Hz) Beta (10–12 Hz)	EEG Data: Baseline Verbal recall task Power—Discrete Fourier Transform (DFT).	Differences in theta and alpha responses during encoding and retrieval. Differences in theta, alpha, and beta responses in high and low performers.
Endedijk et al. (2017)	29 participants (19 F/10 M) Age: (M = 52.48 months, SD = 1.94) Mu (7–12 Hz) Beta (16–20 Hz)	The color-naming task. Action observation task Cooperation task (double-tube task). The entrainment task.	4-year-old children who exhibited greater motor system engagement during action observation, indicated by reduced beta-power, demonstrated higher success in early peer cooperation.
Liao et al. (2015)	21 participants: 11 toddlers (7 F/4 M) Age: (M = 41 months, SD = 4) 10 mothers’ age: (M:35.5) Mu (7–9 Hz) Beta (15–18 Hz)	Component power spectral density (PSD). Component event-related spectral perturbations (ERSPs). IC clustering.	Age-matched head models indicated that mu sources were located in both the left and right sensorimotor cortex. When children observed their parents’ actions, there was a noticeable suppression of power in the mu (7–9 Hz) and beta (15–18 Hz) frequency ranges. Remarkably, the children’s mu suppression during action observation mirrored adult patterns in all observed properties.
Belalov et al. (2014)	91 participants: 41 orphans (14 F/27 M) Age: (M = 36 months, SD = 2) 50 children raised in a family (19 F/31 M) Age: (M = 35 months, SD = 3) Theta (4–6 Hz) Alpha (7–10 Hz) Beta (11–29 Hz) Gamma (30–45 Hz)	EEG was recorded in three situations: Spontaneous EEG (eyes open): 60 s; while the child listened to a short poem: 20 s; while the child heard meaningless speech: 20 s. Spectral power density (SPD). Tests: The Language Scale of the Bayley Scales of Infant and Toddler Development III (BSID-III).	The Bayley assessment showed that children raised in orphanages had significant delays in language development compared to those raised in families. During the speech stimulus, alpha oscillations become desynchronized, while theta, beta, and gamma oscillations show increased synchronization. Children raised in families showed notable increases in gamma rhythm SPDs across 13 leads in both hemispheres. Conversely, orphans exhibited this increase in only 8 locations, predominantly localized in the left hemisphere.
Schneider et al. (2016)	36 participants: 18 children (9 F/9 M) Age: (M = 10.94, SD = 0.94) 18 adults (9 F/9 M) Age: (M = 24.41, SD = 4.37) ERP (P600, N400) Theta (4–8 Hz) Beta (12–30 Hz)	Grammaticality judgment task. Event-related spectral perturbations (ERSPs). Fourier power spectra density (PSD).	Unlike adults, children exhibited a distinct pattern with a noticeable N400 effect and did not show a decrease in beta or theta activity in response to grammatical violations.
Orekhova et al. (2001)	60 participants Age: (M = 37.3 rd week, SD = 1.96) Alpha (6.4–10 Hz)	EEG: Attention to an object in the visual field peek-a-boo game. Behavioral data: the total time of anticipatory attention was calculated across all 10 trials.	Infants who displayed longer periods of anticipatory attention had higher absolute spectral amplitude across a wide frequency range in both experimental tasks. Alpha synchronization in the posterior parietal cortex actively inhibits certain networks involved in maintaining attention to the peripheral visual field, rather than representing a passive or idle state.
Orekhova et al. (2006)	47 participants: 28 infants (9 F/19 M) Age: (M = 9.2, SD = 1.3) 19 children (8 F/11 M) Age: (M = 5 years 5 months, SD = 359 days) Theta (3.6–5.6 Hz) Mu rhythms (6.0–8.8 Hz)	EEG was recorded during baseline (visual attention) and two test conditions: exploration of toys (manipulation), and attention to ‘social’ stimulation (speech). Event-related spectral power.	Theta responses increase in both infants and children during attention-requiring test conditions, with infants showing a broader topographical increase compared to children. (a) Theta frequency was observed at 3.6–5.6 Hz in the posterior temporal region and occipital–parietal–temporal regions of infants under attention conditions. (b) Theta frequency in the frontal region of children is observed to be in the range of 4–7.6 Hz.
Bell et al. (2007)	50 participants (22 F/28 M) Age: (8 months, when the 25 children were 4.5 years of age, they joined the research again) 6–9 Hz Alpha (8–13 Hz)	Spontaneous EEG. Discrete Fourier transform (DFT) Coherence. Infant working memory/inhibitory control task. The Day-Night Stroop-like task. The Yes-No task.	(a) In infancy, working memory was associated with widespread changes in EEG power and coherence across the entire scalp, indicating broad cortical involvement. (b) By early childhood, these associations were more localized, focusing primarily on medial frontal areas for EEG power and involving specific pairs like medial frontal/posterior temporal, and medial frontal/occipital for EEG coherence.
Blankenship et al. (2018)	242 participants (121 F/121 M) Age: (M = 6.64 years, SD = 0.44) Theta (4–7 Hz) Alpha (8–13 Hz)	Episodic memory (retrieval) tasks. Working memory tasks (A backward digit span (BDS) task). Tests: Woodcock–Johnson (WJ) III The Peabody Picture Vocabulary Test IV.	EEG alpha power in frontal regions and theta power in temporal regions during the working memory task were used to statistically predict both math and reading abilities.
Güntekin et al. (2020)	34 participants: 16 children Age: (M = 6.69 years, SD = 0.48) 18 young adults Age (M = 21.32 years, SD = 3.28) Theta (4–7 Hz) Alpha (8–13 Hz)	The Boston Naming Test (BNT). Free and Cued Selective Reminding Test (FCSRT). Tests: Wechsler Intelligence Scale for Children, Fourth Edition (WISC-IV).	Frontocentral theta and alpha phase-locking play a crucial role in brain maturation and the achievement of successful memory performance. Young adults had higher theta and alpha phase-locking than children over the frontal and central locations. Children exhibited heightened theta phase-locking and increased left alpha power in response to remembered objects compared to forgotten objects. The children had higher parietal–occipital alpha phase-locking than the young adults.
Rajan et al. (2021)	29 participants (19 F/10) Age: (M = 6.10 years, SD = 0.26) Theta (4–7 Hz)	Memory binding task. Discrete Fourier transform (DFT). Natural log (In) transform.	Theta rhythms play a significant role in memory-binding processes during middle childhood.
Stroganova et al. (2006)	44 participants Age: (M = 19.6 weeks; SD= 2.59) Theta (3.6–5.2 Hz)	The attention tasks (ExA, EnA, and CA). Spectral analysis (Fast Fourier Transform). Spectral theta peak amplitude (SA). Tests: Bayley-2 scale	At five months of age, children who maintain attention on a hidden object demonstrate the emergence of a highly synchronized theta rhythm compared to their child stages.
Gomarus et al. (2006)	18 participants (4 F/15 M) Age: (M = 10.4 years, SD = 1.5) ERPs Theta (4–8 Hz) Alpha (8–12 Hz).	Selective memory search task ERD, ERS.	Theta ERS was most evident during the most challenging task condition in the recognition set, while alpha ERD exhibited a load effect only during memorization.
Spironelli et al. (2010)	70 participants: 28 children (14 F/14 M) Age: (M = 10.01 years, SD = 0.18) 22 young adults (11 F/11 M) Age: (M = 22.59 years, SD = 3.67) 20 middle-aged adults (7 F/13 M) Age: (M = 59.10 years, SD = 7.11) Delta (1–4 Hz) Theta (4–8 Hz) Alpha (8–13 Hz Beta (β1: 13–20 Hz, β2: 21–28 Hz)	Three linguistic tasks (orthographic, phonological, semantic). Spectral FFT analysis.	In adults, task-dependent right lateralization was observed in theta and alpha distributions, whereas children exhibited differences primarily between linguistic and non-linguistic tasks without clear lateralization patterns.
Jiang et al. (2017)	37 participants (18 F/19 M; 10 excluded) Age: (4.5–5.5 years) Delta (1–3 Hz) Theta (3–8 Hz) Alpha (8–13 Hz) Beta (13–30 Hz) Gamma (30–70 Hz)	Event-related spectral perturbation (ERSP).	Negative stimuli, compared to neutral ones, induced greater theta ERS in children who showed improved cognitive efficiency with negative emotional content.
Morasch et al. (2009)	48 participants (16 F/32 M) Age: (M = 10 months, 6 days) alpha (6–9 Hz)	Discrete Fourier transform (DFT) Tasks: The activity-matched baseline Encoding (modeling) Recall Test.	Infants who demonstrated successful ordered recall exhibited an increase in brain activity from the baseline to the task at the front temporal scalp areas. In contrast, infants who did not show ordered recall had no changes in brain activity from baseline to task.
Schneider et al. (2018)	39 participants: 23 adults (14 F/9 M) (M = 24 years, SD = 4.3) 16 children (8 F/8 M) (M = 10.88 years, SD = 0.96 years) Theta, alpha, and beta = 3–30 Hz	Event-related spectral perturbation.	The increase in children’s theta power after 2400 ms over the frontal and right frontocentral areas during the processing of grammatically and semantically correct sentences significantly differed from that of adults.
Attaheri et al. (2022)	60 participants Age: (longitudinal cohort: (M = 212.2 days SD = 7.2 to M = 333.1 days SD = 5.6)) Delta Theta	(mTRF) method in delta, theta, and alpha bands. Periodogram (PSD) analysis. Phase-amplitude coupling. (PAC) for delta–beta, delta–gamma, theta–beta, and theta–gamma coupling.	(a) PAC was significantly present at all ages, with both delta and theta bands driving the coupling. (b) Delta and theta were significant carrier phases, linking with higher-frequency amplitudes in the infant brain.
Meyer et al. (2023)	23 infants (10 F/13 M) Age: 15.5–16.5 months (M = 15.9) Theta (4–5 Hz)	EEG recorded during demonstration video clips at one of the three conditions (normal, high, and variable) includes five goal-directed movement actions (acting on balls, cups, and rings). After EEG recording, 1 min. exploration phase started and infants had the opportunity to act on the objects themselves. Time–frequency analysis of Fz, FCz, Cz.	Infants with increased frontal theta activity were prone to investigating new objects within the present task framework. Variability has an important role in drawing infants’ attention to and thereby fostering their learning from infant-directed actions (IDAs).
Meyer et al. (2019)	29 children (9 F/10 M) Age: 4 years (M = 52.48, SD = 1.94 months) Theta (3–6 Hz)	Tasks: No task, a color-naming task, an ımitation task. Theta band (3–6 Hz) power was calculated using a Fast Fourier Transform with multitaper method.	Frontocentral theta power was found higher when children engaged in tasks than when they were not involved in tasks. Higher theta power over left frontotemporal electrode sites when being engaged in the language-related task compared to the motor-related task Theta oscillations have a role in top-down control.
Grossmann et al. (2007)	12 infants (5 girls), 4-month-olds Gamma oscillations (ERPs)		
Köster et al. (2019)	38 infants (14 F/24 M) Age: nine-month-olds (M = 9.4 months, SD = 7 days) Theta (3–5 Hz) Alpha (5–7 Hz)	Four classical violations of expectation paradigms. The picture sequences were visually flickered at a theta (4-Hz) or an alpha (6-Hz) frequency. Steady-state visually evoked potentials (SSVEPs) of posterior electrodes (O1, Oz, O2, P3, P4, Pz, P7, P8) were analyzed.	At unexpected events, the visually entrained theta oscillations increased. However, entrained alpha oscillations did not differ between outcomes. Expected and unexpected events are factors that cause a frequency-specific SSVEP response.
Tang et al. (2019)	201 participants (124 F/77 M) 58 children (38 F/20 M) Age: 10–12 years (M = 10.83, SD = 0.82) 64 adolescents (39 F/25 M) Age: 14–17 years (M = 15.02, SD = 0.98) 79 young adults (47 F/32 M) Age: 18–28 (M = 19.42, SD = 1.92) Theta (4–7 Hz)	Social Exclusion Task (participants played the online ball-toss game Cyberball). ERSP between 200 to 600 ms at F3, FCz, P4. ITC from 100 to 400 ms. Event-related theta EEG power and phase coherence.	Adolescents and children showed the highest spectral power at 400–600 ms on the rejection part. However, adults’ spectral power was the highest theta spectral power at the not-my-turn event. Increased theta spectral power responses to both social exclusion and threats of exclusion shown in adolescent development (ITC); adolescents showed lower levels of left frontal theta synchrony in response to rejection compared to children and adults.
Orekhova et al. (1999)	60 infants Age: 7–12 months born after 33.5–41 weeks gestation (M: 37.3, SD = 1.96) Theta 1 (3.6–4.8 Hz) Theta 2 (5.2–6.0 Hz)	EEG recorded in three attention conditions: looking without overt emotional reactions, the peek-a-boo game, and attention to the adult’s reappearance.	EEG theta rhythm (3.6–6 Hz) synchronized during internally controlled (anticipatory) attention. As infants become older, the attention-related slow theta synchronization tends to decrease. The fast theta rhythm appeared only during effortful concentration of attention.
Bazhenova et al. (2007)	16 infants (9 F/7 M) Age: 20 weeks (M = 19.7; SD = 0.89) Theta (3.6–5.6 Hz) Alpha (6.0–9.0 Hz)	EEG recorded an adult’s smiling (SF) and a blank face (BF) in a face-to-face setting.	Looking at the adult’s BF causes increases in theta activity in the 4.8 Hz narrow band, observed over the majority of anterior and right posterior temporal scalp areas. The right hemisphere is more dominant to changes in a partner’s facial expressivity.
Yordanova et al. (2009)	70 participants (37 F/33 M) 54 children (25 F/29 M) 16 young Adults (12 F/4 M) Age: M = 306.4, SD = 55.7 Theta (4–7 Hz) Alpha (8–14 Hz)	Three task conditions: passive listening condition (PLC), simple reaction task (SRT), serial learning reaction task (SLRT). EROs.	Results show that fast alpha oscillations are stronger in SLRT condition and more synchronized in children than adults. In the slower theta-to-alpha range, the phase synchronization increases with age. Developmental dynamics specific for theta, alpha-1, and alpha-2 frequencies differ in memory activation tasks.
Nanova et al. (2018)	36 children (13 F/20 M) Age: 7–10 years Delta (0.5–4 Hz) Theta (4–7 Hz) Slow alpha (7–10 Hz) Fast alpha (10–14 Hz)	Sensorimotor task with fixed stimulus sequences. ERPs N1, P2, N2 and P3 amplitudes. Phase-synchronization of EROs.	Proactive processing mode was marked by increased pre-stimulus theta activity. Notable reduction in the temporal synchronization of event-related theta/alpha oscillations within 300 milliseconds after the stimulus.
Nanova et al. (2011)	36 healthy children (18 F/18 M): Age: 7–8 years old (M = 7.7) (9 F/9 M) Age: 9–10 years old (M = 9.2) (9 F/9 M) Delta (0.5–4 Hz), Theta (4–7 Hz), Slow Alpha(7–10 Hz) Fast Alpha (10–14 Hz)	EEG recorded 3 different conditions: auditory stimulation, a passive listening condition (PLC), a simple reaction task (SRT), and a serial learning reaction task (SLRT). Phase-locking analysis. Auditory ERP.	Accelerated functional maturation of processing networks is frequency-specific and shown in increased girls’ phase-locking of the slower (delta, theta, and slow alpha) but not for faster (fast alpha) frequency bands. Between 7 and 10 years of age, the development of neurophysiological mechanisms crucial for early auditory information processing varies based on gender.
Ashkinazi et al. (2010)	30 healthy children (M = 6.05, SD = 0.75): Age: 5–6 years old (n = 14) Age:6–7 years old (n = 16) Set: a plastic set (n = 14), a rigid set(n = 16) Theta (4–7 Hz) Alpha (8–13 Hz)	Resting-state EEG. Visual set paradigm. Spectral power analysis using FFT.	Higher alpha band spectral power (SP) has been shown at the plastic set rather than the rigid set. Both hemsipheres at occipital lobes showed increased alpha activity.
Chung et al. (2022)	49 full-term infants (25 F/24 M): Age: 21 infants, 9-month-olds (M = 9.21, SD = 10.0 days) (8 F/13 M) Age: 28 infants, 12-month-olds (M = 12.21, SD = 17.8 days) (17 F/11 M) Mu (6- to 9-Hz)	EEG recorded during action observation conditions: grasp condition, cane-use condition. Action execution period: Allow the infant to attempt trials in grasping and using a cane. Interchannel phase coherence (ICPC).	At the 6–9 Hz frequency range phase, coherence of central–ocipital regions is higher than shorter distances such as central–parietal and central–frontal regions while observing grasping action.
Vollebregt et al. (2015)	27 children (16 F/9 M): Age: 7–10 years (M = 9.11, SD = 1.29) Alpha (8–12 Hz)	Attention paradigm (Posner’s cueing paradigm). EEG recorded 2 min EO, 2 min EC, and during the task.	Healthy children exhibited adult-like alpha lateralization patterns during covert attention. Children who were less affected by spatial cueing showed stronger left alpha modulation.
